# COVID-19 lockdown beneficial effects on lung function in a cohort of cystic fibrosis patients

**DOI:** 10.1186/s13052-021-00970-4

**Published:** 2021-01-18

**Authors:** Alessia Giuseppina Servidio, Giulia Capata, Laura Levantino, Guglielmo Riccio, Sarah Contorno, Egidio Barbi, Massimo Maschio

**Affiliations:** 1 University of Trieste, Trieste, Italy; 2 IRCCS Burlo Garofolo, Trieste, Italy

## Introduction

In December 2019, an infectious disease, caused by a novel coronavirus, emerged in Wuhan City, China. The disease was later named coronavirus disease 2019 (COVID-19) and the virus which causes it was named severe acute respiratory syndrome coronavirus 2 (SARS-CoV-2). To limit the spread of the COVID-19 in our country, the Italian Government imposed the first urgent measures on February 23rd, 2020 and then a national quarantine from March 9th, 2020 to May 4th, 2020 restricting the free movement of the entire population to the essential minimum. The presence of chronic diseases has been considered one of the main risk factors for COVID-19-related morbidity and mortality. Cystic fibrosis (CF) is a chronic and life-shortening disease affecting up to 1 in 2000 to 3000 live births among Caucasian population. As respiratory complications are the major cause of morbidity and mortality in CF patients, earlier preventive measures, e.g. home isolation, self-monitoring, reinforcing adherence to therapy, were strongly recommended to reduce the risk of COVID-19 infection in these patients [1]. To avoid unnecessary hospital visits and viral spread, telemedicine was encouraged, routine visits were cancelled and pulmonary function testing was performed only if therapeutic decisions were essential [2].

Home isolation and the implementation of careful hygienic practices limited the spread of seasonal viral or bacterial infection. It is widely acknowledged that, among people with CF, respiratory viruses are associated with prolonged respiratory illness, showing a clear association with pulmonary exacerbations, lung function decline, and risk of death [3, 4].

This study evaluates the impact of home isolation of CF patients during lockdown on admission rates pulmonary, respiratory exacerbations and pulmonary function parameters (in particular, forced expiratory volume in 1st second, FEV1).

## Methods and results

We retrospectively evaluated patients with CF followed at the Regional Centre for the Diagnosis and Treatment of Cystic Fibrosis of the Institute for Maternal and Child Health “Burlo Garofolo” in.

Trieste (Italy). These patients are routinely visited on average four times a year.

Data were collected from November 1st 2019, to June 30th 2020, during clinical visits of CF patients throughout a two-month period before (January to February 2020, “Time 1”) and after (May to June 2020, “Time 2”) the lockdown in Italy (imposed from March 8th to May 4th), in addition to a two-month period from November to December 2019 (“Time 0”) assumed as the baseline. For each patient we analyzed lung function trend comparing data from 2019 to 2020 with those collected from the same months in 2018–2019 (January to February 2019, “Time 1”; May to June 2019, “Time 2” and November to December 2018, “Time 0”).

The study involved 34 of the 93 patients followed in the CF Centre (~ 37% of the total number, 40% among the cooperative ones); all of them were undergoing three clinical visits over the timelines and were able to cooperate with pulmonary function testing. Included patients were 20 females and 14 males and had a median age of 22.5 years (range 8 to 42). We chose not to include patients with CF undergoing lung transplantation (n. 9), since they ordinarily lead a locked-down lifestyle due to their immunosuppressive therapies. Patients undergoing less than two check-ups in the study period were also excluded. Nine out of 34 patients were treated with CFTR modulators (7/10 with lumacaftor/ivacaftor, 1/10 with ivacaftor and 1/10 with elexacaftor/tezacaftor/ivacaftor) and started CFTR modulators before November 2018 except for the only patient in therapy with elexacaftor/tezacaftor/ivacaftor who started therapy in March 2020. For each patient we studied pulmonary function with spirometry, testing FEV1 (volume of exhaled air during a forced breath in the 1st second), FVC (maximal volume of air that can be forcefully expired following maximum inspiration) and FEF 25–75% (mean forced expiratory flow between the 25 and 75% of the FVC) as an estimation of distal airways resistance. Each patient performed a spirometry at least 3 times and in accordance with the standardized Acceptability, Usability and Repeatability Criteria for FEV1 and FVC [5]. Individual characteristics of patients are reported in Table 1.

**Table 1:** Characteristics of patients at baseline in 2019–2020: sex, age, CF genotype and CFTR modulators

Sex	Age	CF genotype	CFTR modulators
F	27	1717-1G- > A/1717-1G- > A	
F	25	F508del/F508del	Lumacaftor/Ivacaftor
F	18	F508del/F508del	Lumacaftor/Ivacaftor
F	9	F508del/R1066H	
F	31	2183AA > G/ 3276C > A	
M	15	F508del/F508del	Lumacaftor/Ivacaftor
M	20	F508del/3002_3003delTG	
F	24	F508del/3849 + 10kbC > T	
M	8	1584 + 18672A > G/F508del	
M	27	N1303K/G542X	
F	14	F508del/F508del	Lumacaftor/Ivacaftor
F	17	G542X/I507del	
F	25	F508del/F508del	Lumacaftor/Ivacaftor
F	16	F508del/621 + 1G- > T	
F	20	F508del/2368-69del11	
M	42	F508del/N1303K	
M	11	F508del/F508del	
M	24	F508del/R1162X	
F	10	I507del/1677delTA	
M	17	F508del/F508del	Lumacaftor/Ivacaftor
M	32	F508del/G542X	
M	36	F508del/I507del	
F	29	F508del/G551D	Ivacaftor
F	30	F508del/CFTRdel17a-18	Elexacaftor/Tezacaftor/ Ivacaftor
F	10	F508del/621 + G	
M	10	F508del/621 + G	
M	34	F508del/CFTRdel17a-18	
F	23	F508del/F508del	Lumacaftor/Ivacaftor
F	21	F508del/G1298X	
M	33	223C > T/UN	
M	22	1717-G > A/R1162X	
F	33	F508del/N1303K	
F	33	F508del/T338I	
F	17	F508del/S466X-R1070Q	

The average difference between baseline parameters collected in 2019–2020 and 2018–2019 can be considered irrelevant. Mean difference at baseline in 2018–2019 compared to 2019–2020 were: − 0.01 (L) and − 2.50 (%) for FVC, 0.00 (L) and − 1.93 (%) for FEV1, 0.04 (L) and 1.30 (%) for FEF 25–75%. Standard Deviations at baseline in 2018–2019 compared to 2019–2020 were: 0.37 (L) and 9.75 (%) for FVC, 0.39 (L) and 12.02 (%) for FEV1, 0.75 (L) and 17.89 (%) for FEF 25–75%.

Each patient was compared at T1 and T2 with baseline (T0) for both years. Data were reported as average differences at T1 and T2 compared with T0.

Referenced data showed an overall improvement of pulmonary function of CF patients from pre-lockdown to post-lockdown period. This improvement was higher than expected on the basis of the trend in 2018/2019. We found the average differences at T1 to be: 0.08 L (0.61%) in 2018–2019 and 0.05 L (1.26%) in 2019–2020 for FVC; 0.12 L (1.93%) in 2018–2019 and 0.04 L (− 0.38%) in 2019–2020 for FEV1; 0.10 L (2.11%) for 2018–2019 and − 0.02 L (− 2.91%) in 2019–2020 for FEF 25–75%. As expected, no statistically significant difference was found at T1 between 2018 and 2019 and 2019–2020.

We found the average differences at T2 to be: 0.03 L (− 0.55%) in 2018–2019 and 0.26 L (6.32%) in 2019–2020 for FVC [(*p* = 0.036 absolute value (L) and *p* = 0.022 for percentage value (%)]; 0.05 L (− 0.72%) in 2018–2019 and 0.24 L (6.09%) in 2019–2020 for FEV1 [(*p* = 0.031 absolute value (L) and *p* = 0.019 for percentage value (%)]; − 0.06 L (− 0.18%) in 2018–2019 and 0.27 L (5.59%) in 2019–2020 for FEF 25–75% [(*p* = 0.039 absolute value (L) and *p* = 0.074 for percentage value (%)].

**Fig. 1:**
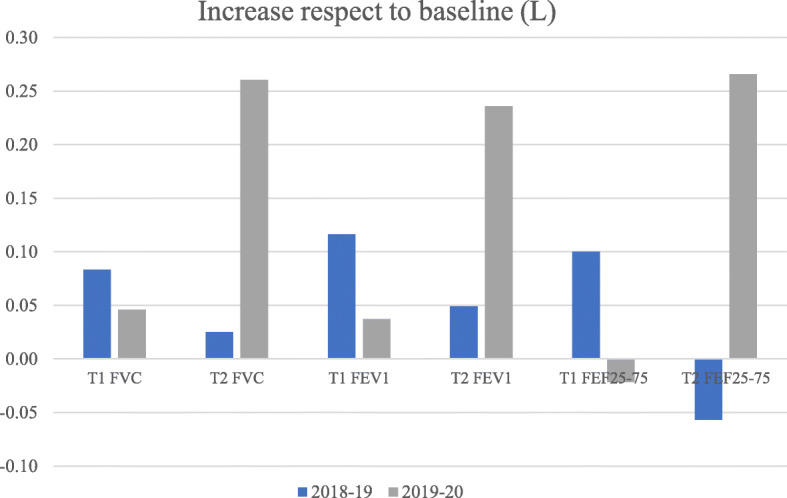
Mean differences in absolute lung function values at T1 and T2 respect to baseline (T0) in 2019–2020 and 2018–2019

Differences for all parameters at T2 were statistically significant except data for FEF 25–75 in percentage value. Summary of results are showed in Fig. 1.

During the lockdown period 6 patients (~ 16%) underwent antibiotic therapies, with admissions for intravenous treatments in 4 cases. During the same study period of the previous 2 years the number of admissions was respectively of 8 in 2018 and 7 in 2019.

**Fig. 2:**
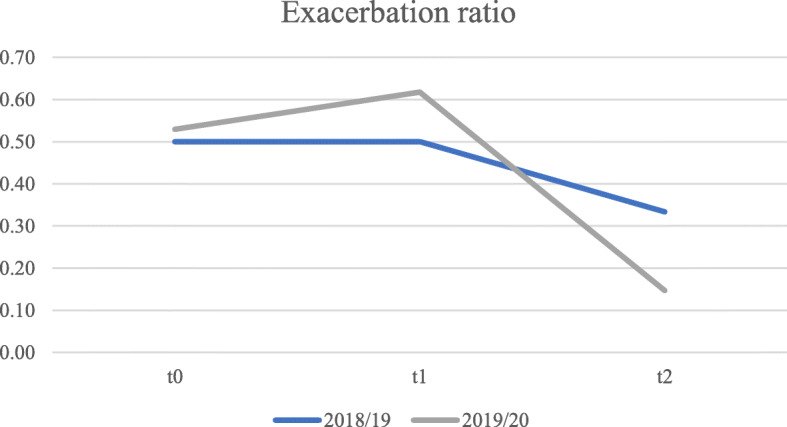
Exacerbation ratio at baseline, T1 and T2 in 2018–2019 and 2019–2020

At baseline the number of exacerbations was similar. Four fewer patients were included in the analysis for 2018–2019 because data were missing. Congruently with results found in spirometric values, we found no statistically significant difference in terms of number of exacerbations comparing data from 2018 to 2019 and 2019–2020 at T1 (14/28 exacerbations in 2018–2019 and 21/34 in 2019–2020), whilst results were statistically significant at T2 (10/30 in 2018–2019 and 5/34 in 2019–2020; *p* = 0.010). Summary of results are showed in Fig. 2.

## Discussion

This study shows that patients with CF benefited from the lockdown in terms of respiratory function, reduced exacerbations and hospital admissions.

In this retrospective series we analyzed the data collected from 34 individuals followed in our CF Regional Center in order to assess the effect of lockdown on pulmonary function parameters.

At the end of the lockdown, we reviewed our patients for routine visits and, among those who had adhered to the restrictive measures, we noticed a marked improvement in clinical conditions and functional lung parameters. Data showed an overall improvement of pulmonary function with a significant average increase of 0.24 L in post-lockdown FEV1 values respect to baseline. This difference was higher than that expected according to the improvement trend noticed in the previous year in the same months (0.05 L).

One obvious reason is that home isolation and implemented hygienic practices prevented the spread of any seasonal viral or bacterial infection in both general and CF population [6]. This is particularly evident in the pediatric population considering that schools and sport activities were closed from the beginning of March in Italy.

A relevant part of daily time in a CF patient is devoted to medications and physiotherapy. This is particularly true as the stage of the disease is more advanced and both medical and physical therapy almost completely occupy time off work or school. As CF patients were forced to stay at home often without attending school, working or attending everyday activities, they may have devoted much longer time in the day to physiotherapy and medications. The consequent increase in treatment compliance might have produced a beneficial effect on respiratory function which was objectified by the increase of post-lockdown spirometric parameters.

While the number of ward admissions was too small to allow a statistical analysis, it was substantially halved during the lock-down period compared to the previous year. Lockdown measures also helped reduce the number of exacerbations respect to those expected when compared with the previous year (10/30 patients in 2018/2019 and 5/34 patients in 2019/2020).

Since lockdown in Italy was lifted in May and daily activities were slowly allowed again, there might have been an increase in viral and bacterial infections in our CF patients However, a baseline fitness of our CF patients due to a better adherence to therapy and caution to the spread of infection might help them to maintain a good pulmonary function in the late post-lockdown phase.

Beside the setting of an exceptional and catastrophic pandemic related lockdown these data suggest that selected patients, e.g. those waiting for a lung transplant, may benefit from a period of isolation and dedicated rest from everyday activities.

## Conclusions

Pulmonary function parameters in CF patients followed in our Center significantly improved in post-lockdown compared to pre-lockdown phase due to home isolation, careful hygienic practices and higher therapeutic compliance. While lockdown is a hopefully non reproducible artificial setting, this evidence suggests that in highly selected cases, e.g. patients waiting for a transplant, a strategy of isolation and increased compliance may offer some benefits. Further data are needed to confirm this hypothesis.
